# *Undaria pinnatifida* Fucoidan-Rich Extract Recovers Immunity of Immunosuppressed Mice

**DOI:** 10.4014/jmb.1908.08026

**Published:** 2019-12-15

**Authors:** Hwan Hee Lee, Yoo Jin Cho, Gun-Hee Kim, Hyosun Cho

**Affiliations:** 1 Department of Pharmacy, Duksung Women’s University, Seoul 0369, Republic of Korea; 2 Duksung Innovative Drug Center, Duksung Women’s University, Seoul 01369, Republic of Korea; 3 Department of Food and Nutrition, Duksung Women's University, Seoul 0169, Republic of Korea

**Keywords:** *Undaria pinnatifida*, fucoidan, cyclophosphamide, T cell immunity, NK cells

## Abstract

We investigated the immune restoration activity of *Undaria pinnatifida* fucoidan-rich extract in cyclophosphamide-induced immunosuppressed mice. C57BL/6 mice were intraperitoneally injected with 80 mg/kg of cyclophosphamide (CP) and orally administered with either drinking water (DW), red ginseng extract (RG), or one of three different doses of *Undaria pinnatifida* fucoidan-rich extract (DSU02 50, 100, and 150 mg/kg). After 14 days, liver, spleen, and whole blood were isolated from each animal. The frequencies of NK and CD3^+^, CD4^+^, and CD8^+^ T cells were significantly increased in splenocytes isolated from the DSU02 100 mg/kg and DSU02 150 mg/kg groups (NK1.1+, 5.4% or 4.9% vs 3.8%; CD3^+^, 39.3% or 37.9% vs 32.3%; CD4^+^, 22% or 20.2% vs 17.4%; CD8^+^, 12.7% or 11.6% vs 10.1%). NK cytotoxicity was enhanced in the DSU02-fed groups at all doses (CP-treated DW, 93.4%; RG, 107.2%; DSU02 50, 107.3%; DSU02 100, 107.3%; DSU02 150, 107.1%), and the proliferation of T cells (CD3^+^, CD4^+^, and CD8^+^) was also greater in the DSU02 100 mg/kg and DSU02 150 mg/kg administered groups compared with the unfed group. Plasma concentrations of TNF-α, IgM, and total IgG from the DSU02 150 mg/kg group were also significantly higher compared with the other groups (TNF- α: CP-treated DW - 21.5 pg/ml, DSU02 150 - 47.1 pg/ml; IgM: CP-treated DW - 82.9 ng/ml, DSU02 150 - 110.8 ng/ml; total IgG: CP-treated DW - 114.4 ng/ml, DSU02 150 - 162.7 ng/ml). We suggest that *Undaria pinnatifida* fucoidan-rich extract could be a promising candidate for a marine natural immune stimulator.

## Introduction

Cyclophosphamide (CP) is known to disrupt DNA replication and inhibit cell proliferation [1, 2]. Thus, CP is widely used as a chemotherapeutic reagent for various cancers including lymphoma, leukemia, breast cancer, and small cell lung cancer [3]. Chemotherapy generally results in severe immunosuppression that lowers the quality of life and increases the risk of infectious diseases [4]. In particular, CP was reported to suppress immune responses by decreasing the number of lymphocytes [5]. Therefore, improving host immunity is very important in cancer treatment, and functional foods with immune stimulatory properties have been explored for many years [6, 7].

Fucoidans, which are sulfated polysaccharides and mostly found in marine brown algae and seaweed, are widely used as an ingredient in dietary supplements. Most fucoidans contain sulfated polysaccharides and fucose with additional sugar constituents such as mannose, galactose, glucose, xylose, and uronic acids [8]. Fucoidans are known to have a wide range of biological activities such as immune stimulatory, anti-viral, and antitumor activity [9-11]. Because polysaccharide-rich compounds are known to serve as strong immune stimulators, the immune-activating effect of fucoidans has attracted much interest [12]. Therefore, the immune-enhancing effect of fucoidans, fucoidan-rich extracts, or fucoidan-combined substances has been extensively studied to fight cancer or infection [13, 14]. Recently, fucoidan was evaluated as a potent adjuvant in a vaccine against *Bordetella bronchiseptica* [13]. In addition, fucoidan extracts from *Undaria pinnatifida* or *Fucus vesiculosus* have been tested as immune boosters in preclinical evaluation of safety in cancer treatment [14].

Fucoidan from the seaweed *Chordaria flagelliformis* showed a similar level of activity with recombinant granulocyte colony-stimulating factor in terms of stimulating hemato-poiesis after cyclophosphamide immunosuppression in mice [15]. The same group also reported that chemically modified fucoidan and related sulfated oligosaccharides significantly induced hematopoiesis in immunosuppressed animals [16]. However, the immune restoration effect of fucoidans or fucoidan-combined extracts in CP-induced immunosuppressed animals has never been investigated.

In this study, we prepared fucoidan-rich extracts from Korean *Undaria pinnatifida* that inhabits Wando-gun, Republic of Korea. *Undaria pinnatifida*-derived fucoidan was reported to have a higher sulfate and L-fucose content, which guarantees a broader range of biological activities, than fucoidans from other origins [17]. We examined the immune-enhancing effect of Korean *Undaria pinnatifida*-derived fucoidan in CP-treated immunosuppressed mice.

## Materials and Methods

### Preparation of *Undaria pinnatifida* Fucoidan-Rich Extract

*Undaria pinnatifida* was collected in April 2017 from Wando-gun, Korea. *Undaria pinnatifida* was washed repeatedly three times with purified water to remove salt and air-dried for 12 h. Dried *Undaria pinnatifida* powder was extracted by acid hydrolysis with 0.1 N HCl (v/v) diluted 10-fold for 8h at 45°C. The hydrolysate was filtered using a decanter and then the supernatant was collected. The collected supernatant was neutralized with 1N NaOH (pH 6-8) and then evaporated to one-tenth of the initial volume. The same volume of 95% (v/v) ethanol was added to the supernatant and soaked for 8h. The extract was filtered with a continuous-centrifuge to collect the pellet. Lyophilized pellet was obtained as an *Undaria pinnatifida* fucoidan-rich powder (yield 25% (w/w)), and the average molecular weight of fucoidan in *Undaria pinnatifida* extract was 200 kDa. The extracted intact fucoidans mostly consisted of carbohydrates (49.5%) with protein (5.2%), salt (1.4%), water (2.98%), and other (13.3%) (provided by Natural endotech Co., Ltd., Korea).

### Induction of Immunosuppression in Mice by Cyclophosphamide (CP)

All the animal experiments were conducted in accordance with the recommendations in the National Research Council’s Guide for the Care and Use of Laboratory Animals. The experimental protocol was approved by the Animal Experiments Committee of Duksung Women’s University (permit number: 2018-003-008). C57/BL6 mice (female, 5 weeks old) were obtained from RaonBio Co. Ltd. (Korea). Mice were adapted to the animal laboratory for 7 days before the experiment and maintained under a controlled temperature (23 ± 2°C) with a 12-h/12-h light/dark cycle. Mice were randomly divided into six groups (*n* = 8) and treated with intraperitoneal injection of 80 mg/kg of cyclophosphamide (CP), excluding the negative control group (NC), once at day 1 and once at day 3.

### Oral Administration of *Undaria pinnatifida* Fucoidan-Rich Extract

The day after two rounds of cyclophosphamide treatment, mice were orally administered with either drinking water, red ginseng extract (RG), or one of three different doses of *Undaria pinnatifida* fucoidan-rich extract (DSU02 50 mg/kg, 100 mg/kg, or 150 mg/kg) for 2 weeks. Body weight was monitored during treatment and organs such as liver and spleen were isolated from each animal on day 14 at the experimental end point. The percentages of NK cells and subpopulations (CD3+, CD4+, or CD8+) of T cells from spleen were measured. Also, whole blood was drawn by cardiac puncture, and plasma cytokines (TNF-α, IFN-γ) or antibodies (IgM, total IgG) were measured.

### Fluorescent Antibody and Cell Surface Antigen Staining

Splenocytes were stained with Mouse Anti-Mouse NK 1.1- APC (BD Biosciences, USA), Rat Anti-Mouse CD3-PE (BD Biosciences, USA), Rat Anti-Mouse CD4-PE (BD Biosciences, USA), and Rat Anti-Mouse CD8-APC (BD Biosciences) according to the manufacturer’s instructions. After staining, cells were analyzed by flow cytometry (Novocyte Flow Cytometer, ACEA Biosciences, USA). The positivity of CD8, CD4, and CD3 was determined by comparison with the defined cutoff values obtained with unstained control cells as previously described [18].

### NK Cytotoxicity Assay

Primary splenocytes were harvested from each animal and the cytotoxic effect of NK cells onto YAC-1 was performed using a CytoTox96 Non-radioactive Cytotoxicity Assay Kit (Promega, USA). Briefly, YAC-1 cells as target cell were plated at a density of 5 × 10^5^ cells per well in a 96-well bottom plate and incubated overnight. The target cells (T) were cocultured with effector cells (**E**), splenocytes, at an E/T ratio of 1:5 for 4 h. The cells were centrifuged at 1,600 ×*g* for 4 min, and the supernatants were transferred per well of a fresh plate. Then, CytoTox96 reagent was added to each well and incubated for 30 min at room temperature in the dark, and finally stop solution was added to each well. Subsequently, absorbance was detected at 490 nm within 1 h using a microplate reader (BMG Labtech, Germany).

### Cell Proliferation Assay 

Cell proliferative effect of *Undaria pinnatifida* fucoidan-rich extract on primary mouse splenocytes was assessed using a Cell Counting Kit-8 (CCK-8, Dojindo, Japan). Cells were seeded at a density of 1 × 10^4^ cells per each well in a 96-well flat bottom plate with or without Con A (1 μg/ml) and incubated for 72 h. Then, CCK-8 solution was added to each well, and the cells were incubated in accordance with the reactive time of the solution. The absorbance was measured using a microplate reader (BMG Labtech,) at 450 nm.

### Proliferation of CD3^+^, CD4^+^, and CD8^+^ T Cells by Carboxyfluorescein Diacetate Succinimidyl Ester (CFSE)

T cell proliferation was assessed using a CellTrace CFSE Cell Proliferation Kit (ThermoFisher, USA). Briefly, the lymphocyte cells were isolated from spleen of C57BL/6 mouse, and then the cells were stained with CFSE (5 μM) staining solution diluted in DPBS for 20 min in a 37°C water bath. The CFSE-stained cells were added to RPMI1640 (Corning, USA) media with 10% heat-inactivated fetal bovine serum (FBS; Korea) and 100 U/ml penicillin and streptomycin (Gibco, USA) and then incubated for 5 min. Next, the cells were centrifuged at 300 ×*g* for 5 min, the supernatants were aspirated, and CFSE-stained cells were plated with a density of 1 × 10^7^ cell per each well in a 6-well bottom plate and incubated for 5 days in 5% CO_2_ at 37°C. After 5 days, the cells were stained with Rat Anti-Mouse CD3-PE (BD Biosciences, NJ, USA), Rat Anti-Mouse CD4-PE (BD Biosciences, USA), and Rat Anti-Mouse CD8-APC (BD Biosciences). The stained cells were analyzed by flow cytometry (Novocyte Flow Cytometer, ACEA Biosciences, USA).

### ELISA Assay

Cell-free supernatants were harvested to measure the production of TNF-α, IFN-γ, IgM, or IgG using a mouse enzyme-linked immunosorbent assay kit (BD Biosciences). Absorbance was measured at 450 nm using a microplate reader (BMG Labtech).

### Statistical Analysis

Data were processed using Microsoft Excel, and the results are presented as mean ± standard deviation (SD). Comparison of means was performed with a t-test or one-way analysis of variance followed by Fisher’s Least Significant Difference as a post hoc test. Differences among groups were considered significant at a value of *p* < 0.05.

## Results

### Effect of *Undaria pinnatifida* Fucoidan-Rich Extract (DSU02) on Body or Organ Weight Changes in Cyclophosphamide (CP)-Treated Immunosuppressed Mice

**Fig. 1:**
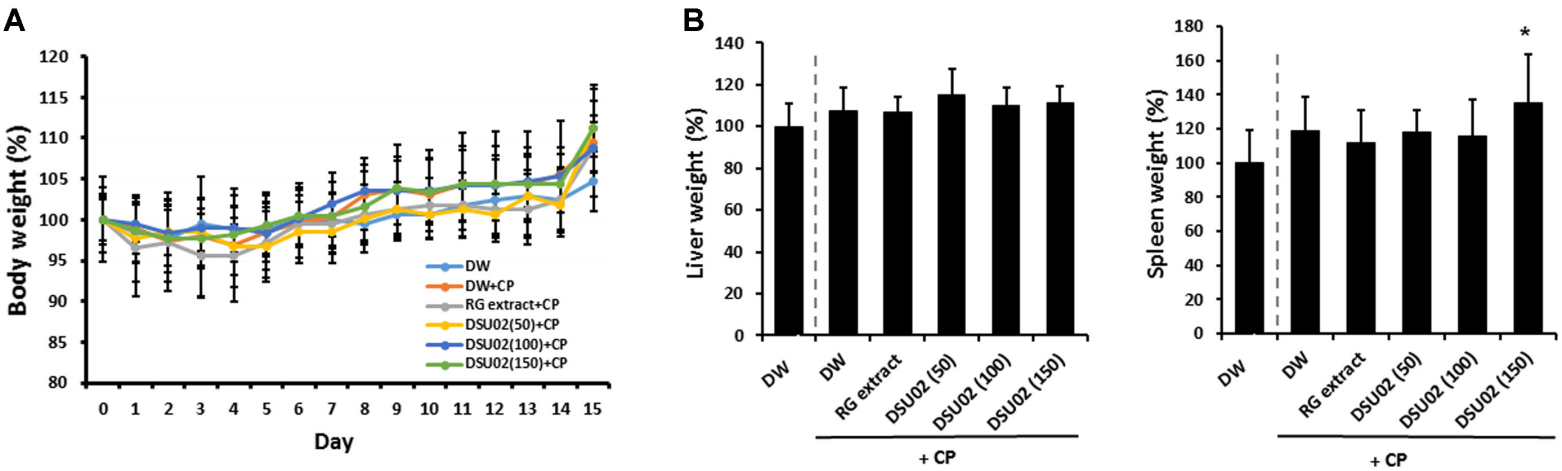
Effect of *Undaria pinnatifida* fucoidan-rich extract (DSU02) on body or organ weight changes in cyclophosphamide (CP)- treated immunosuppressed mice. Mice were intraperitoneally injected with 80 mg/kg of cyclophosphamide (CP) and subsequently administered with either drinking water (DW), RG extract (10 mg/kg), or one of three different doses of *Undaria pinnatifida* fucoidan-rich extract (DSU02 50 mg/kg, 100 mg/kg, 150 mg/kg). (**A**) Whole body weight change was monitored daily until the experimental end point. (**B**) Liver or spleen weight changes. Significant difference is shown: *p < 0.05.

Mice were randomly divided into six groups (*n* = 8) and treated with intraperitoneal injection of 80 mg/kg of cyclophosphamide (CP), excluding the negative control group, once at day 1 and once at day 3. The day after two rounds of cyclophosphamide treatment, mice started to be orally administered with either drinking water, red ginseng extract (RG), or one of three different doses of *Undaria pinnatifida* fucoidan-rich extract (DSU02 50 mg/kg, 100 mg/kg, or 150 mg/kg) for 2 weeks to investigate the immune-enhancing effect of *Undaria pinnatifida* fucoidan-rich extract in immunosuppressed mouse models. The body weight changes were checked daily until the end of the experiment. In addition, liver and spleen were removed and weighed at the experimental end point. A steady increase in whole body weight in all groups was recorded through the experimental period, which indicates that there is no general toxicity by oral administration of *Undaria pinnatifida* fucoidan-rich extract (DSU02) (Fig. 1A). Spleen weight increased more in the *Undaria pinnatifida* fucoidan-rich extract (DSU02) 150 mg/kg-fed group, to 135%, compared with the normal drinking water group (100%) or the CP-treated drinking water group (118%) (Fig. 1B).

### Effect of *Undaria pinnatifida* Fucoidan-Rich Extract (DSU02) on the Frequency and Cytotoxicity of NK Cells in CP-Treated Immunosuppressed Mice

**Fig. 2:**
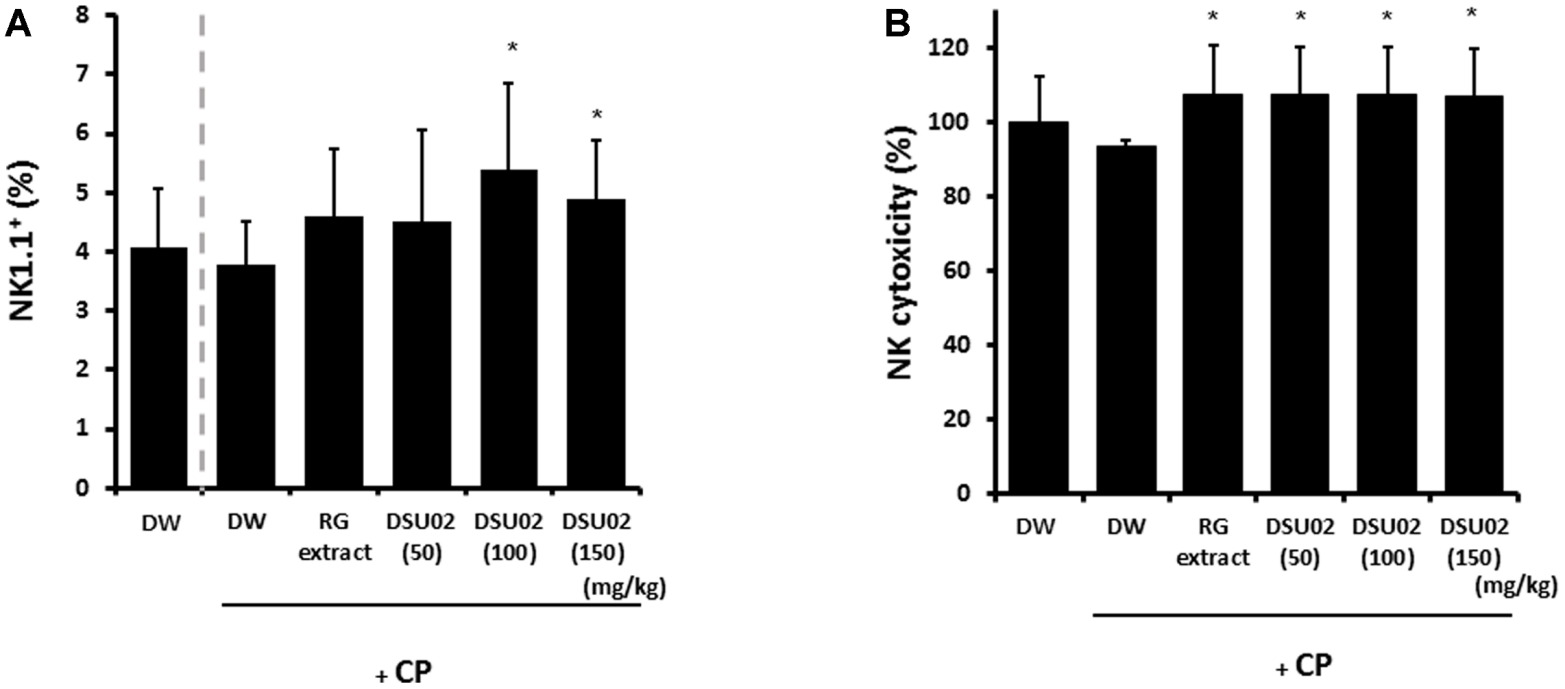
Effect of *Undaria pinnatifida* fucoidan-rich extract (DSU02) on the frequency and cytotoxicity of NK cells in CP-treated immunosuppressed mice. Mice were intraperitoneally injected with 80 mg/kg of cyclophosphamide (CP) and subsequently administered with either drinking water (DW), RG extract (10 mg/kg), or one of three different doses of *Undaria pinnatifida* fucoidan-rich extract (DSU02 50 mg/kg, 100 mg/kg, 150 mg/kg). Spleen was isolated from each animal on day 14 after treatment. Single cell suspension of splenocytes was prepared and the percentages of (**A**) NK1.1+ and (**B**) NK cytotoxicity were analyzed using flow cytometry. Significant difference is shown: *p < 0.05.

Spleen was isolated individually from each group of animals on day 14 after treatment. Single cell suspension of splenocytes was prepared and the frequency of NK cells and cytotoxicity were determined. The frequency of NK1.1^+^ cells showed a significant increase from 3.8% in the CP-treated DW group up to 5.4% and 4.9% in the DSU02 100 mg/kg and 150 mg/kg-fed groups, respectively (Fig. 2A). In the case of NK cytotoxicity against Yac1 cells, all administered groups, namely the RG extract- and DSU02-fed groups, showed a significant increase (RG, 107.2%; DSU02 50, 107.3%; DSU02 100, 107.3%; DSU02 150, 107.1%) compared with the unfed immunosuppressed groups (DW group or CP-treated DW group) (Fig. 2B).

### Effect of *Undaria pinnatifida* Fucoidan-Rich Extract (DSU02) on the Frequency of T Cell Subpopulations (CD3+, CD4+, and CD8+ T cells) in CP-Treated Immunosuppressed Mice

**Fig. 3:**
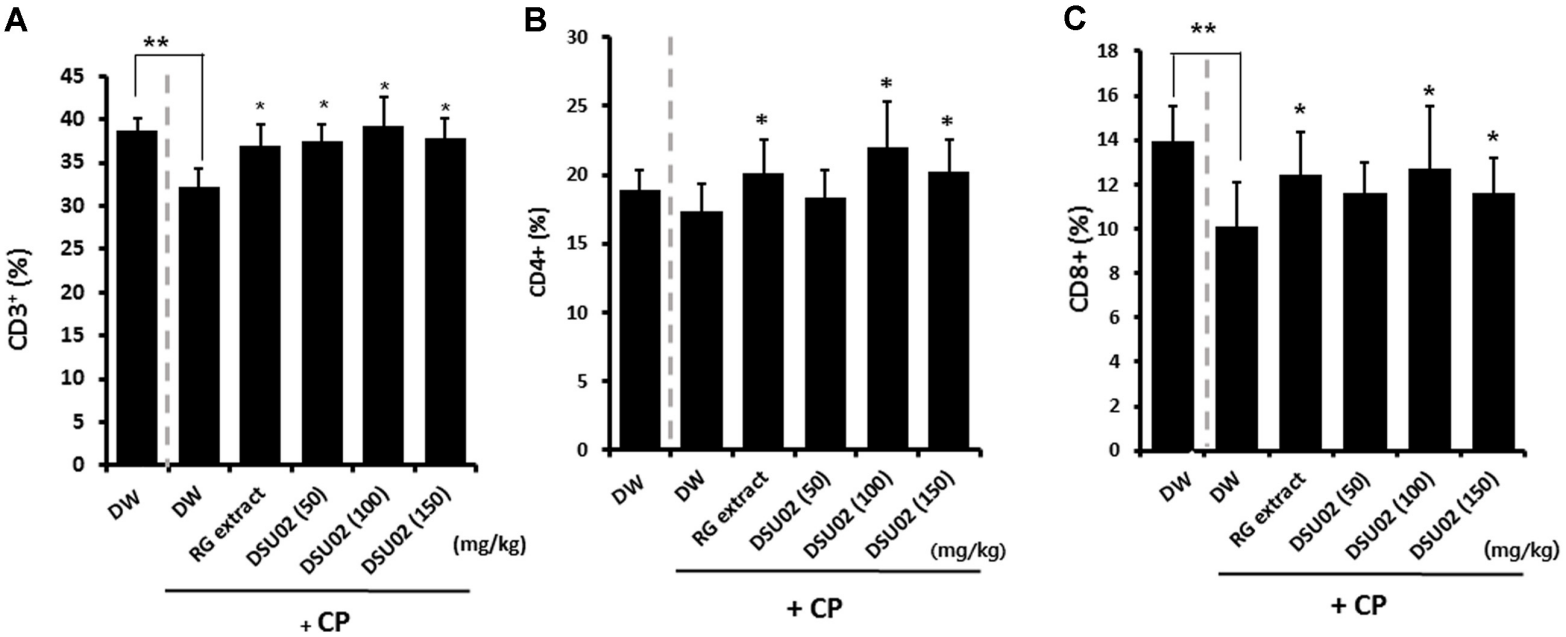
Effect of *Undaria pinnatifida* fucoidan-rich extract (DSU02) on the frequency of T cell populations in CP-treated immunosuppressed mice. Mice were intraperitoneally injected with 80 mg/kg of cyclophosphamide (CP) and subsequently administered with either drinking water (DW), RG extract (10 mg/kg), or one of three different doses of *Undaria pinnatifida* fucoidan-rich extract (DSU02 50 mg/kg, 100 mg/kg, 150 mg/kg). Spleen was isolated from each animal on day 14 after treatment. Single cell suspension of splenocytes was prepared and the percentages of (**A**) CD3+, (**B**) CD4+, and (**C**) CD8+ were analyzed using flow cytometry. Significant difference is shown: ** or * *p* < 0.05.

The percentages of CD3^+^, CD4^+^, and CD8^+^ T cells were also determined using a flow cytometer. The percentage of CD3^+^ T cells showed a significant decrease from 38.7% to 32.3% in the cyclophosphamide-induced immunosuppressed group (CP-treated DW) compared with the immunocompetent group (DW) (Fig. 3A). However, the frequency of CD3^+^ T cells recovered up to a high of 39.3% in the RG extract- and DSU02-fed groups (RG, 37%; DSU02 50, 37.4%; DSU02 100, 39.3%; DSU02 150, 37.9%), which is a similar pattern as that with the frequency of CD8^+^ T cells (DW, 14% vs CP-treated DW, 10.1% vs RG, 12.4%; DSU02 50, 11.6%; DSU02 100, 12.7%; DSU02 150, 11.6%) (Figs. 3A and 3C). In the case of CD4^+^ T cells, there was no significant reduction in frequency in the immunosuppressed group (CP-treated DW) compared with the immunocompetent group (DW), although the frequency was higher in the RG extract- and DSU02 100 and 150-fed groups (DW, 18.9% vs CP-treated DW, 17.4%vs RG, 20.1%; DSU02 50, 18.3%; DSU02 100, 22%; DSU02 150, 20.2%) (Fig. 3B).

### Effect of *Undaria pinnatifida* Fucoidan-Rich Extract (DSU02) on the Proliferation of Whole Splenocytes, CD3+, CD4+, and CD8+ T Cells, in CP-Treated Immunosuppressed Mice

**Fig. 4:**
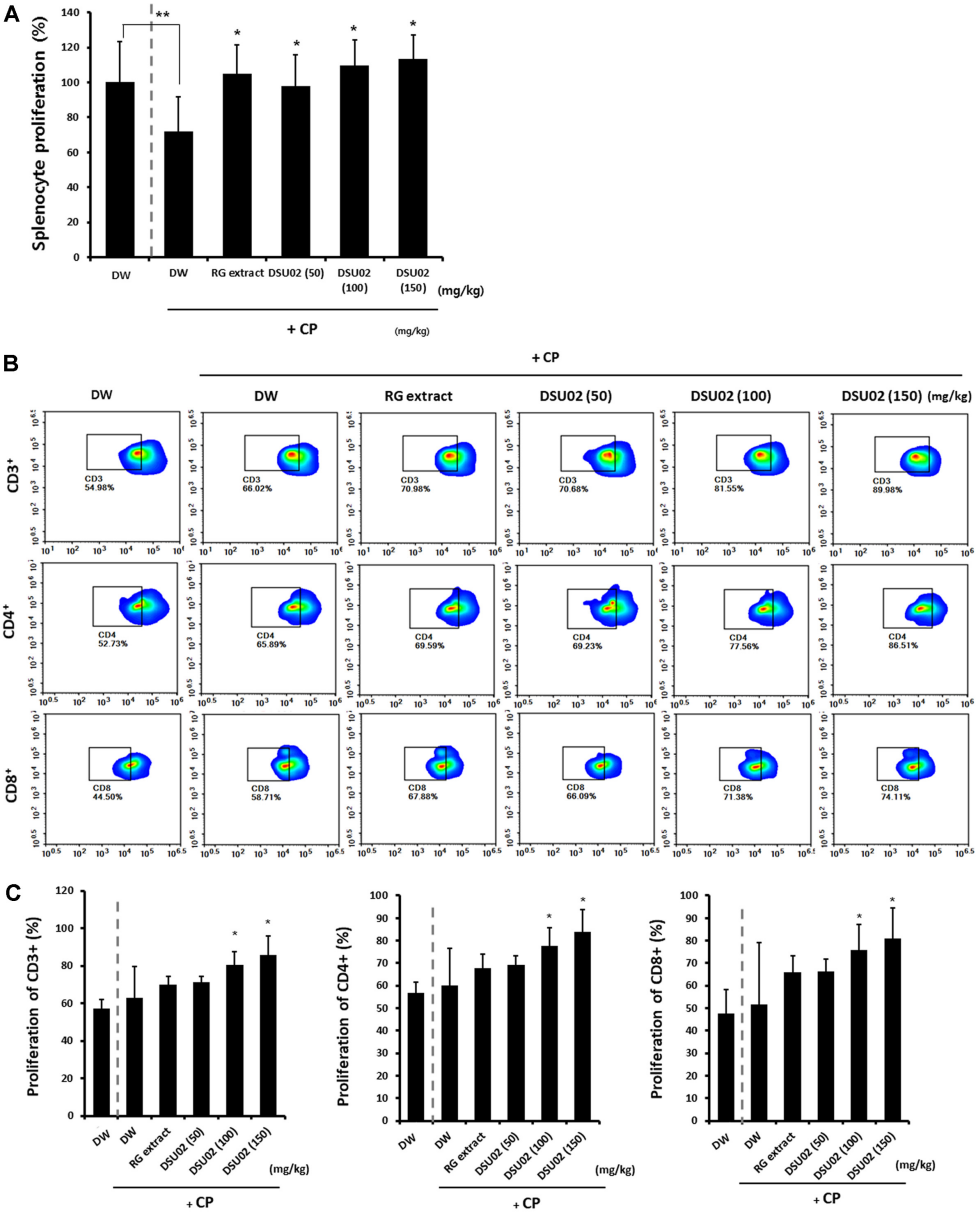
Effect of *Undaria pinnatifida* fucoidan-rich extract (DSU02) on the proliferation of whole splenocytes, CD3+, CD4+, and CD8+ T cells, in CP-treated immunosuppressed mice. Mice were intraperitoneally injected with 80 mg/kg of cyclophosphamide (CP) and subsequently administered with either drinking water (DW), RG extract (10 mg/kg), or one of three different doses of *Undaria pinnatifida* fucoidan-rich extract (DSU02 50 mg/kg, 100 mg/kg, 150 mg/kg). Primary splenocytes were isolated from each animal and stimulated with Con A (1 μg/ml ). (**A**) The proliferation of splenocytes was measured by CCK-8 assay at 72 h after incubation. Single cell suspension of splenocytes from each animal was stained with CFSE and cultured for another 7 days. (**B**) Representative FACS plot of CD3+, CD4+, and CD8+ expression on T cells. (**C**) The specific proliferations of CD3+, CD4+, and CD8+ were analyzed using flow cytometry. Significant difference is shown: ** or * *p* < 0.05.

Spleen was removed from each group of animals on day 14 after treatment. Single cell suspension of splenocytes was prepared and the proliferation of whole splenocytes was measured at 72 h after incubation with concanavalin A (ConA) (1 μg/ml). The proliferation of splenocytes from the cyclophosphamide-treated group (CP-treated DW) was significantly suppressed compared with that from the immunocompetent group (DW) (DW, 100% vs CP-treated DW, 71.8%); however, the RG extract- and all DSU02-treated groups (50 mg/kg, 100 mg/kg, 150 mg/kg) induced significant proliferation of splenocytes (RG, 104.8%; DSU02 50, 98.1%; DSU02 100, 109.5%; DSU02 150, 113.3%) (Fig. 4A).

We next investigated which kinds of T cell populations from the RG extract- or DSU02-fed groups contributed to the induction of splenocyte proliferation. A single cell suspension of splenocytes was stained with CFSE and cultured for another 7 days. In addition, the specific proliferations of CD3^+^, CD4^+^, and CD8^+^ T cells were analyzed using a flow cytometer. The results showed that there was significant proliferation of both CD4^+^ (DW, 56.7%; CP-treated DW, 60%; RG, 67.8%; DSU02 50, 69.2%; DSU02 100, 77.7%; DSU02 150, 84%) and CD8^+^ T cells (DW, 47.6%; CP-treated DW, 51.5%; RG, 66%; DSU02 50, 66.3%; DSU02 100, 75.9%; DSU02 150, 80.8%) in the DSU02 100 mg/kg and 150 mg/kg-fed groups (Figs. 4B and 4C).

### Effect of *Undaria pinnatifida* Fucoidan-Rich Extract (DSU02) on the Production of TNF-α, IFN-γ, and Antibodies (IgM, Total IgG) in CP-Treated Immunosuppressed Mice

**Fig. 5:**
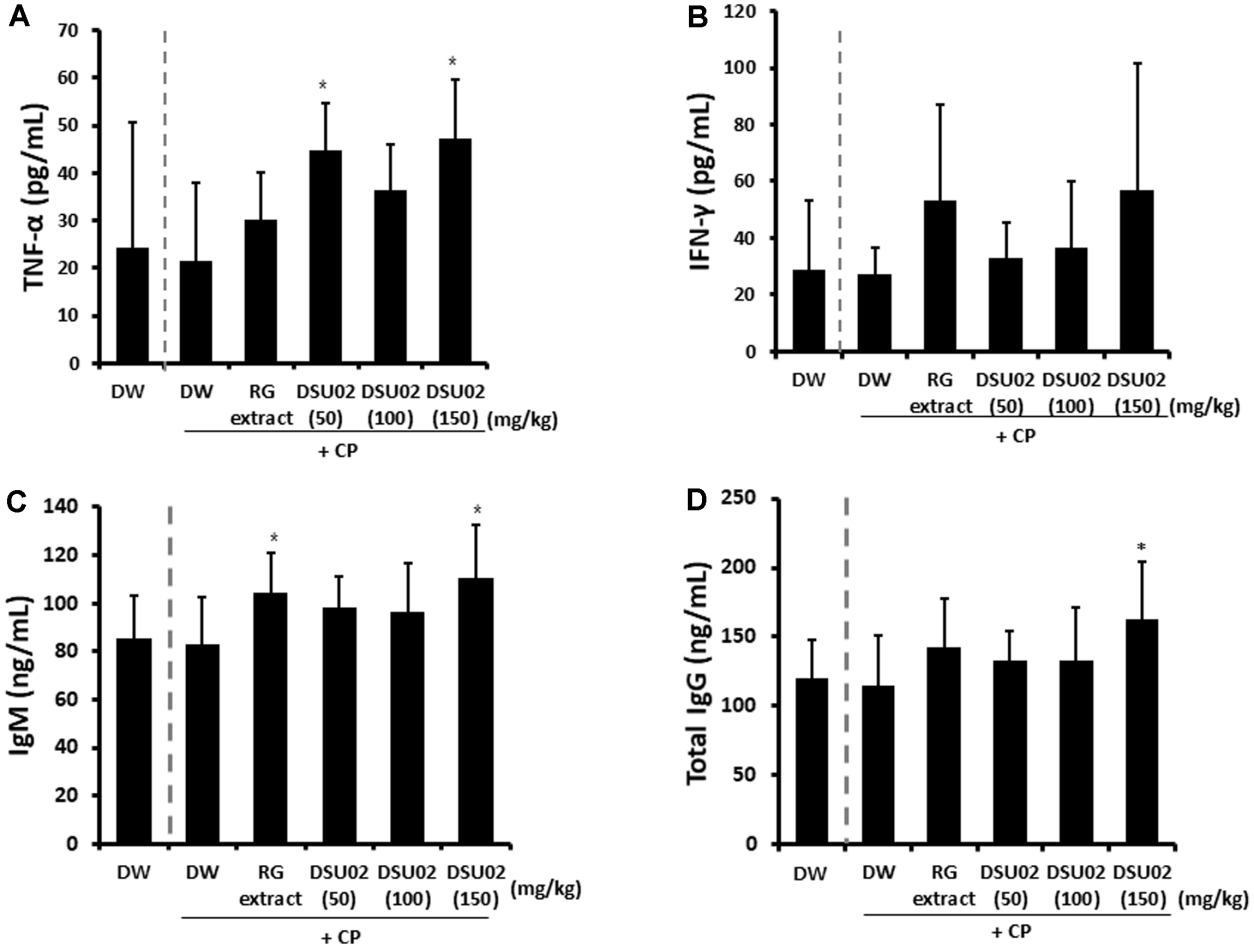
Effect of *Undaria pinnatifida* fucoidan-rich extract (DSU02) on the production of TNF-α, IFN-γ, IgM, and total IgG in CPtreated immunosuppressed mice. Mice were intraperitoneally injected with 80 mg/kg of cyclophosphamide (CP) and subsequently administered with either drinking water (DW), RG extract (10 mg/kg), or one of three different doses of *Undaria pinnatifida* fucoidan-rich extract (DSU02 50 mg/kg, 100 mg/kg, 150 mg/kg). Whole blood was drawn at the experimental end point and the productions of TNF-α (**A**), IFN-γ (**B**), IgM (**C**), and total IgG (**D**) were measured using ELISA. Significant difference is shown: *p < 0.05.

Whole blood was drawn on day 14 after treatment, and the amounts of immune activating cytokines (TNF-α, IFN-γ) as well as plasma antibodies (IgM, total IgG) were measured using ELISA. TNF-α was significantly induced from the DSU02 50 mg/kg and 150 mg/kg-fed groups (CP-treated DW, 21.5 pg/ml; DSU02 50, 44.8 pg/ml; DSU02 150, 47.1 pg/ml) compared with the other groups (Fig. 5A). However, there was no notable increase in the production of IFN-γ in any DSU02-fed group (Fig. 5B). Subsequently, the amounts of plasma IgM and total IgG from each animal were measured, and there was a significant increase in plasma IgM from 82.9 ng/ml in the CP-treated DW group up to 104.5 ng/ml and 110.8 ng/ml for the RG extract and DSU02 150 mg/kg groups, respectively (Fig. 5C). In the case of total IgG, only the DSU02 150 mg/kg group recorded a significant increase in the amount (162.7 ng/ml) compared with the other groups (Fig. 5D).

## Discussion

In this study, we explored the immune restoration activity of *Undaria pinnatifida* fucoidan-rich extract (DSU02) in cyclophosphamide-induced immunosuppressed mice. Cyclophosphamide (CP)-treated mice were orally administered with either drinking water (DW), red ginseng extract (RG), or one of three different doses of *Undaria pinnatifida* fucoidan-rich extract (DSU02 50, 100, and 150 mg/kg) for 14 days. The frequencies of NK and CD3^+^, CD4^+^, and CD8^+^ T cells from each group were compared and the functional activation of lymphocyte populations was investigated. Many studies have reported the immunostimulatory effect of fucoidan or fucoidan-combined extract in vitro as well as in vivo. Jin group reported that *Undaria pinnatifida*-derived fucoidan induced the production of IL-6, IL-8, and TNF-α in human neutrophils through the activation of the PI3/AKT signaling pathway [19]. Also, the sulfate or acetyl groups of fucoidan were shown to contribute to the activation of mouse macrophages, Raw 264.7 cells, by stimulating the production of NO, TNF-α, and IL-6 [20, 21]. Vetvicka group also found that a higher amount of fucoidan content provided a greater proliferative effect on lymphocytes such as T cells and B cells [22]. Recently, we found that *Undaria pinnatifida* fucoidan-rich extract stimulated Raw 264.7 cells to produce significant nitric oxide metabolites and cytokines (TNF-α, IL-1α, IL-1β, and IL-6) and induced the proliferation of splenocytes through the activation of pERK protein [23]. We also reported that oral administration of *Undaria pinnatifida* fucoidan-rich extract significantly increased the activation of T cells and the production of TNF-α, IFN-γ, and IgM in C57BL/6 mice [23]. Therefore, our previous study proposed a comprehensive immune-stimulatory effect of *Undaria pinnatifida* fucoidan-rich extract in vitro as well as in vivo.

However, some groups reported an anti-inflammatory effect of fucodians or fucoidan-rich extracts that seems to be associated with a relatively lower composition of carbohydrates or contamination of fucosterol [24, 25]. The biological activities of fucoidans or fucoidan-rich extracts are known to be different depending on the types of raw material for the extract, carbohydrate content, or combinations with other substances [22, 26].

Applications of cytostatic chemotherapy or immuno-suppressant reagents in cancer or organ transplantation lead to deterioration of the immune system, which results in a higher risk of infection, inhibition of hematopoiesis, etc. A few studies have reported a hematopoietic effect of fucoidans or fucoidan-combined molecules in immuno-suppressed animal model [15, 16]. Anisimova group recently reported that fucoidan from the seaweed *Chordaria flagelliformis* induced significant neutropoiesis, thrombopoiesis, and erythropoiesis in CP-treated animals, which suggests that fucoidan could rebuild the immune system in severe immunosuppression [16].

However, the direct immune restoration activity of fucoidans or combined extracts has not been investigated yet in immunosuppressed animals. Our study is the first report that *Undaria pinnatifida* fucoidan-rich extract (DSU02) significantly induces immune activation in CP-treated immunosuppressed mouse model. We initially found that CP treatment induced significant immunosuppression by reduction in the frequencies of CD3^+^ and CD8^+^ T cells and inhibition of splenocyte proliferation, although there was no difference between the CP-treated group and untreated group in terms of whole body or organ weight changes (Figs. 3A, 3B, 3C, and 4A). Subsequently, oral administration of RG extract or *Undaria pinnatifida* fucoidan-rich extract (DSU02) certainly recovered the frequencies of all T cell populations (CD3^+^, CD4^+^, CD8^+^) and the proliferative capacity of splenocytes (Figs. 3A, 3B, 3C, and 4A). In particular, the proliferation of T cell subpopulations (CD3^+^, CD4^+^, CD8^+^) in splenocytes was predominantly increased in the DSU02 100 mg/kg or 150 mg/kg administered groups compared with other groups (Figs. 4B and 4C). However, *Undaria pinnatifida* fucoidan-rich extract (DSU02) had no effect on either the frequency or the proliferative capacity of a B (B220^+^) cell population (data not shown), which implies that *Undaria pinnatifida* fucoidan-rich extract (DSU02) seems to target T cells rather than B cells for immune activation.

In vivo injection of *Undaria pinnatifida*-derived fucoidan increased the frequency of NK cells, and these NK cells showed significant cytotoxicity against YAC-1 cells as well as the secretion of IFN-γ [26]. Although we were not able to observe the activation of NK cells in normal C57BL/6 mice (data not shown), we showed that oral administration of *Undaria pinnatifida* fucoidan-rich extract (DSU02) induced an increase of NK cell frequency along with cytotoxic activity (Figs. 2A and 2B).

The results show that only TNF-α, and not IFN-γ, was significantly increased in the *Undaria pinnatifida* fucoidanrich extract (DSU02 50, 150 mg/kg) administered group, which suggests that the immunostimulatory effect of *Undaria pinnatifida* fucoidan-rich extract (DSU02) is not enough to induce the production of IFN-γ in immuno-compromised mouse model (Figs. 5A and 5B). However, oral administration of *Undaria pinnatifida* fucoidan-rich extract (DSU02) 150 mg/kg induced successfully both IgM and total IgG in plasma of CP-treated immunosuppressed mice (Figs. 5C and 5D).

In summary, we are the first to report that *Undaria pinnatifida* fucoidan-rich extract has the capability of regaining the normal level of immunity in CP-induced immunosuppressed animals, which is strongly associated with an increase in cell frequency as well as functional activation of NK cells and T cells. We suggest that *Undaria pinnatifida* fucoidan-rich extract could be an immune booster in immunosuppressed anti-cancer therapy.

## References

[1] Brode S, Cooke A (2008). Immune-potentiating effects of the chemotherapeutic drug cyclophosphamide. Crit. Rev. Immunol..

[2] Colvin OM (1999). An overview of cyclophosphamide development and clinical applications. Curr. Pharm. Des..

[3] Galluzzi L, Buqué A, Kepp O, Zitvogel L, Kroemer G (2015). Immunological effects of conventional chemotherapy and targeted anticancer agents. Cancer Cell.

[4] Shirani K, Hassani FV, Razavi-Azarkhiavi K, Heidari S, Zanjani BR, Karimi G (2015). Phytotrapy of cyclophosphamide-induced immunosuppression. Environ. Toxicol. Pharmacol..

[5] Huyan XH, Lin YP, Gao T, Chen RY, Fan YM (2011). Immunosuppressive effect of cyclophosphamide on white blood cells and lymphocyte subpopulations from peripheral blood of Balb/c mice. Int. Immunopharmacol..

[6] Chirumbolo S (2012). Plant phytochemicals as new potential drugs for immune disorders and cancer therapy: really a promising path?. J. Sci. Food Agric..

[7] Abuajah CI, Ogbonna AC, Osuji CM (2015). Functional components and medicinal properties of food: a review. J. Food Sci. Technol..

[8] Maria IB, Anatolii IU (2008). Structural analysis of fucoidans. Nat. Prod. Commun..

[9] Ponce NM, Pujol CA, Damonte EB, Flores ML, Stortz CA (2003). Fucoidans from the brown seaweed Adenocystis utricularis: extraction methods, antiviral activity and structural studies. Carbohydr. Res..

[10] Maruyama H, Tamauchi H, Iizuka M, Nakano T (2006). The role of NK cells in antitumor activity of dietary fucoidan from *Undaria pinnatifida* sporophylls (Mekabu). Planta Med..

[11] Lean QY, Eri RD, Fitton JH, Patel RP, Gueven N (2015). Fucoidan extracts ameliorate acute colitis. PLoS One.

[12] Tzianabos AO (2000). Polysaccharide immunomodulators as therapeutic agents: structural aspects and biologic function. Clin. Microbiol. Rev..

[13] Kim SY, Joo HG (2015). Evaluation of adjuvant effects of fucoidan for improving vaccine efficacy. J. Vet. Sci..

[14] Mathew L, Burney M, Gaikwad A, Nyshadham P, Nugent EK, Gonzalez A (2017). Preclinical evaluation of safety of fucoidan extracts from *Undaria pinnatifida* and *Fucus vesiculosus* for use in cancer treatment. Integr. Cancer Ther..

[15] Anisimova N, Ustyuzhanina N, Bilan M, Donenko F, Usov A, Kiselevskiy M (2017). Fucoidan and fucosylated chondroitin sulfate stimulate hematopoiesis in cyclophosphamide-induced mice. Mar. Drugs.

[16] Anisimova NY, Ustyuzhanina NE, Bilan MI, Donenko FV, Ushakova NA, Usov AI (2018). Influence of modified fucoidan and related sulfated oligosaccharides on hematopoiesis in cyclophosphamide-induced mice. Mar. Drugs.

[17] Mak W, Wang SK, Liu T, Hamid N, Li Y, Lu J (2014). Anti-proliferation potential and content of fucoidan extracted from sporophyll of new Zealand *Undaria pinnatifida*. Front Nutr..

[18] Lee HH, Kang H, Cho H (2017). Recovery of NK(CD56+CD3-) cells after one year of tenofovir therapy for chronic Hepatitis B infection. J. Microbiol. Biotechnol..

[19] Jin JO, Yu Q (2015). Fucoidan delays apoptosis and induces pro-inflammatory cytokine production in human neutrophils. Int. J. Biol. Macromol..

[20] Teruya T, Tatemoto H, Konishi T, Tako M (2009). Structural characteristics and in vitro macrophage activation of acetyl fucoidan from Cladosiphon okamuranus. Glycoconj J..

[21] Teruya T, Takeda S, Tamaki Y, Tako M (2010). Fucoidan isolated from laminaria angustata var. longissima induced macrophage activation. Biosci.Biotechnol. Biochem..

[22] Vetvicka V, Vetvickova J (2017). Fucoidans stimulate immune reaction and suppress cancer growth. Anticancer Res..

[23] Lee HH, Cho YJ, Yu D, Chung D, Kim GH, Kang H (2019). *Undaria pinnatifida* fucoidan-rich extract induces both innate and adaptive immune responses. Nat. Prod. Commun..

[24] Sanjeewa KK, Fernando IP, Kim EA, Ahn G, Jee Y, Jeon YJ (2017). Anti-inflammatory activity of a sulfated polysaccharide isolated from an enzymatic digest of brown seaweed Sargassum horneri in RAW 264.7 cells. Nutr. Res. Pract..

[25] Lee SH, Ko CI, Ahn G, You S, Kim JS, Heu MS (2012). Molecular characteristics and anti-inflammatory activity of the fucoidan extracted from Ecklonia cava. Carbohydr. Polym..

[26] Zhang W, Oda T, Yu Q, Jin Jo (2015). Fucoidan from Macrocystis pyrifera has powerful immune-modulatory effects compared to three other fucoidans. Mar. Drugs.

